# Chemometric Tools to Point Out Benchmarks and Chromophores in Pigments through Spectroscopic Data Analyses

**DOI:** 10.3390/molecules27010163

**Published:** 2021-12-28

**Authors:** Giulia Festa, Claudia Scatigno, Francesco Armetta, Maria Luisa Saladino, Veronica Ciaramitaro, Viviana Mollica Nardo, Rosina Celeste Ponterio

**Affiliations:** 1 CREF-Museo Storico Della Fisica e Centro Studi e Ricerche “Enrico Fermi”, Via Panisperna 89 a, c/o Piazza del Viminale 1, I-00189 Roma, Italy; giulia.festa@cref.it; 2 Dipartimento Scienze e Tecnologie Biologiche, Chimiche e Farmaceutiche-STEBICEF and INSTM UdR-Palermo, Università di Palermo, Viale Delle Scienze Bld. 17, I-90128 Palermo, Italy; marialuisa.saladino@unipa.it (M.L.S.); ciaramitaroveronica@gmail.com (V.C.); 3 CNR-Istituto per i Processi Chimico, Viale Ferdinando Stagno d’Alcontres 37, I-98158 Messina, Italy; mollica@ipcf.cnr.it (V.M.N.); ponterio@ipcf.cnr.it (R.C.P.)

**Keywords:** ancient pigments, spectroscopic techniques, chemometrics discrimination, elemental and molecular benchmarks

## Abstract

Spectral preprocessing data and chemometric tools are analytical methods widely applied in several scientific contexts i.e., in archaeometric applications. A systematic classification of natural powdered pigments of organic and inorganic nature through Principal Component Analysis with a multi-instruments spectroscopic study is presented here. The methodology allows the access to elementary and molecular unique benchmarks to guide and speed up the identification of an unknown pigment and its recipe. This study is conducted on a set of 48 powdered pigments and tested on a real-case sample from the wall painting in *S. Maria Delle Palate di Tusa* (Messina, Italy). Four spectroscopic techniques (X-ray Fluorescence, Raman, Attenuated Total Reflectance and Total Reflectance Infrared Spectroscopies) and six different spectrometers are tested to evaluate the impact of different setups. The novelty of the work is to use a systematic approach on this initial dataset using the entire spectroscopic energy range without any windows selection to solve problems linked with the manipulation of large analytes/materials to find an indistinct property of one or more spectral bands opening new frontiers in the dataset spectroscopic analyses.

## 1. Introduction

Classification of different types of pigments extracting the characteristic variables with chemometrics and non-destructive spectroscopic techniques are the new trend scenario [1,2]. X-ray fluorescence (XRF) and Fourier transform infrared spectroscopy (FTIR) stand out [3]. Significant shifts of the characteristics bands from pigments made it a challenge to interpret the spectra [3]. 

In the case of painted surfaces [4], micro-sampling is required to confirm the type of the pigment by additional destructive techniques or model samples to easily compare the data [5]. In this framework, a lot of parameters need to be considered such as the raw materials [6,7,8,9,10,11], the specific technique and the specific setup [12,13]. In the real case additional parameters such as the effects of binders and the degradation processes contribute to the final spectrum [14]. Their composition provides complex spectrum in which the interpretation of spectral features for extraction of information would be ambiguous [14]. 

Spectroscopic data pre-processing [15,16] together with projection methods using chemometric tools, such as PCA, is an analytical approach applied in different spectroscopic techniques [17,18,19]. 

Pre-treatments and data fusion are quite important: dataset are submitted to peak alignment [2], normalization processes or a band area selection and processed with regression analysis [20], observing the separation through PCA, linear discriminant analysis or cluster analysis [21]. 

Here, the entire spectral region (frequency domain) of a large and heterogeneous historical pigments’ dataset (48 natural powdered pigments, organic and inorganic) is selected for the purpose of pattern recognition by complementary characterization techniques such as ED-XRF, Raman and FTIR spectroscopies and different set-ups visualizing all the elemental and molecular contributions in accordance with the variance associate of the PCA. In this sense, the intensity of the frequency together with the explained variance reveal the spectral benchmarks, the main features of the entire dataset to distinguish the elemental markers and vibration modes, classifying the pigments and to find unambiguous benchmarks, useful to the spectra comparison. To verify the methodology, a real-case sample comes from the wall painting in *St Maria Delle Palate di Tusa* (Messina, Italy) and previously analysed [22] is here successfully tested confirming the elemental nature, a Fe-based pigment, and therefore its colour. Indeed, the real-case sample is classified as ochre [22]. The suggested PCA discrimination analysis (both elemental and molecular) can help the pigments’ identification on a painting characterised by different colours’ layers.

## 2. Materials and Methods

### 2.1. Historical Pigments

**Figure 1:**
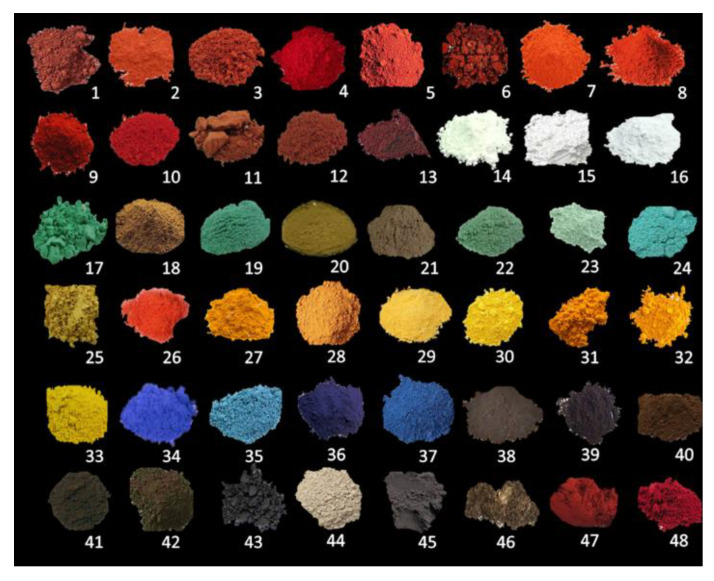
Set of 48 natural powdered pigments by Zecchi store collection. From 1 to 13, Red pigments: Hematite, *Ercolano*, *Cinabrese*, Carmine, Ochre, Dragon’s blood, Coral, Red Lead, Alizarin, Cinnabar, *Bolo*, Jasper, *Morellone*; from 14 to 16, White Pigments: White Lead, *San Giovanni*, White Bone; from 17 to 33, Earths (green and yellow pigments): *Malachite*, *Campeggio*, Chrysocolla, Aloe, Brazilwood, Jasper, *Verdaccio*, Verdigris, *Arzica*, Saffron, Turmeric, Yellow Ochre, *Giallorino*, Yellow *Napoli*, *Gommagutta*, Orpiment, Stil de grain; from 34 to 37, Blue pigments: Smalt, Azurite, Indigo, Lapis lazuli; from 38 to 43, Black Lamp, Ivory, Sepia, Bitumen, Vine Black, and Graphite; from 44 to 46: *Vicenza* Earth, *Romana* Earth, and Schist. Finally, the lake pigment, *Gommalacca* and *Garanza*, 47 and 48 respectively. The natural pigments reported in Italics are pure of Italian origin (e.g., *Ercolano* red is obtained from iron-deposits near *Ercolano*, Naples).

A set of 48 natural powdered pigments (Figure 1, Table S1) by Zecchi catalogue is investigated using 4 spectroscopic techniques and 6 different spectrometers through likewise instrument set-ups obtaining: (a) elemental characterization via the ED-XRF analysis; (b) functional groups identification via Raman and Infrared spectroscopy per a total of 384 spectra. The natural pigments are used as a reference for a real case sample: they are modern samples because they are very close to the composition of historical pigments and therefore constitute a good dataset comparison. To validate the methodology for archaeological matrixes where the pigments are mixed with binders and are subject to degradation processes, a real-case sample is here tested (see Section 2.2). 

### 2.2. St Maria Delle Palate di Tusa

**Figure 2:**
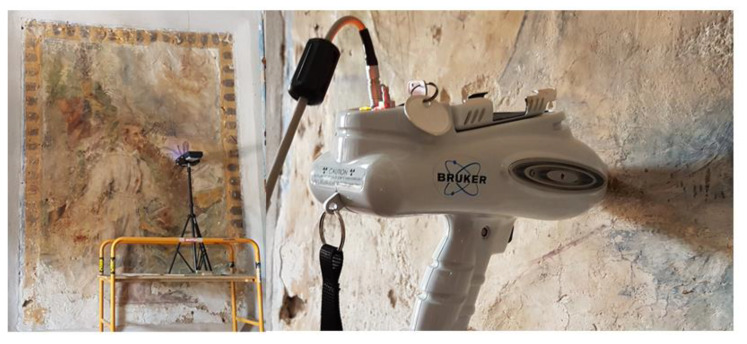
Wall painting of St. Francis inside Church of *S. Maria Delle Palate* during the measurement’s campaign.

The church of *St Maria Delle Palate*, located in the municipality of *Tusa*, in the province of Messina (Italy) is the result of rebuilding on the remains of the Norman church, dated to 1551, with two successive consolidations occurred between 1734 and 1753, under the bishop *Giacomo Bonanno*, and in 1978, made by the *Genio Civile* of Messina. Inside the church a wall painting, probably representing St. Francis in the act of receiving the stigmata, is saved (Figure 2). A combined use of complementary Raman and XRF spectroscopies (through portable instrumentations) has been applied to investigate the pigments of the wall painting by analysing the principal-coloured area [22]. The XRF spectrum of red area (acquired with the same Bruker set-up) is here used for the validation of the proposed methodology.

### 2.3. X-ray Fluorescence Spectroscopy (XRF)

Two different XRF portable instruments are used: XRaman and Tracer III SD AXS. 

XRaman is an innovative portable spectrometer designed to carry out in-situ, fast, and non-destructive combined elemental, and molecular analyses, by the complementary of ED-XRF and Raman techniques. EDXRF and Raman techniques are integrated into a compact detection head and the coincidence of the analysis area is always under control. The system works in a complete contactless mode with an optimal focus distance of about 1 cm from the sample. The XRF system is fully integrated into the compact detection head equipped with a large area fast Silicon Drift Detector (SDD). The SDD with an active area of 25 mm^2^ and energy resolution <135 eV measured on the MnKα line (5.890 eV) allow the detection of the elements with atomic number Z > 11 with input photon rate up to 100,000 counts per second. The excitation source is a transmission X-Ray generator, operating at 5–200 μA and 10–50 kV, Rh anode, max 8 W, microfocus (400–800 μm). Three automatically software selectable collimators (1 mm, 2 mm, 4 mm) and four automatically software selectable X-Ray primary beam filters are implemented. The special design of the developed probe for cultural heritage applications results in an analysis area of 80–1000 μm diameter, and it strongly reduces the laser power density on the sample, regardless of the distance to it (in the operational range). In this work, the XRF spectra are acquired for 50 s, in the range of 30–50 keV and a current of 20 μA. 

Tracer III SD Bruker AXS portable spectrometer has a Rhodium Target X-Ray tube operating at 40 kV and 11 mA and the detection of fluorescence X-rays by a 10 mm^2^ silicon drift X-Flash detector allows the detection of elements with atomic number Z > 11 with a resolution approximately of 145 eV at 200,000 cps. A window of 3–4 mm in diameter determines the sampled area. Each spectrum is acquired for 30 s. The S1PXRF^®^ software is used for data acquisition and spectral assignments.

### 2.4. Infrared Spectroscopy

The Attenuated Total Reflectance (ATR) spectra are recorded by using two instruments: Vertex 70 and Micro FT-IR LUMOS spectrometers. The Vertex 70 Advanced Research FT-IR Spectrometer equipped with Platinum ATR and diamond crystal (n = 2.4) are used to acquire ATR spectra in the 70–4000 cm^−1^ range, with a step of 2 cm^−1^. The measurements are carried out with 200 scans at 2 hPa. In the case of low absorption samples, the 2000–2200 cm^−1^ range shows the characteristic auto-absorption feature of the diamond crystal. 

The μ-FTIR LUMOS spectrometer equipped with a germanium crystal (n = 4.0) is used to acquire ATR spectra in the 600–4000 cm^−1^ range, with a step of 2 cm^−1^. 

The Total Reflectance (TR) Infrared spectra are recorded by using two instruments: Alpha and μ-FTIR LUMOS spectrophotometers. The Alpha spectrometer is used to acquire TR spectra in the range of 360–4000 cm^−1^, with a step of 2 cm^−1^. The measurements are carried out with 200 scans on flat surface pigment pellets ~5 mm prepared by pressing a few mg of pigment allowing an easy and reproducible acquisition of the spectra.

The μ-FTIR LUMOS spectrometer is used to acquire TR spectra in the range of 600–4000 cm^−1^ with a step of 2 cm^−1^. The instrument allows to carry out measurements in several configurations such as transmission, ATR, and reflection.

### 2.5. Raman Spectroscopy 

The Raman spectroscopy measurements are performed by two instruments: BRAVO and XRaman. A next-generation handheld Raman Spectrometer (BRAVO) is used to collect the Raman spectra. In many cases, raw material validation by Raman spectroscopy is not allowed due to fluorescence phenomena. The instrument uses SSE^TM^ (Sequentially Shifted Excitation) patented fluorescence mitigation that permits the measuring of a more extensive range of materials with handheld Raman than ever before. BRAVO is equipped with two excitation lasers (DuoLaser™ Excitation) having wavelengths centred at 785 and 853 nm working together to mitigate the fluorescence phenomena and offers the highest sensitivity across the entire spectral range. The spectra are collected in the 300–3200 cm^−1^ range in automatic set-up mode. Automatic set-up guarantees for each spectrum repeatability and accuracy. The spectra are acquired on solid pigments without sample manipulation. The BRAVO thanks to the Dual LASER^TM^ excitation and the SSE^TM^ can acquire the spectra with the highest sensitivity mitigating the fluorescence simultaneously.

The XRaman system is composed of a probe integrated into the detection head and an external unit with a high-performance CCD spectrometer and a stabilized laser. The instrument uses an excitation laser at 785 nm and the analysis range is 110–3000 cm^−1^. A Fiber Optic spectrometer is optimized for high performance Raman spectroscopy with 2048-pixel, 3 stage-TE cooled and regulated CCD detector. The probe is integrated into the detection head with a 100–200 μm excitation fiber and 200–400 μm read fiber. The spectra are collected in the 2–3666 cm^−1^ range with the following parameters: whites [time = 4 s, frames = 50, laser power = 80 mW], ochres [time = 4 s, frames = 20, laser power = 50 mW], earth colours, green, blue, and black ones [time = 5 s, frames = 20, laser power = 50 mW].

### 2.6. Pre-Data-Treatment

**XRF data treatment**. Initially, 31 elements have been identified (Table S2 of the Supplementary). Of these, 20 elements are selected removing the “fixed signals”, such as the Rh (Kα1 20.21 keV, Lα1 2.69 keV), Ar (Kα1 2.95 keV), the Ni (Kα1 7.48 keV), and Pd (Kα1 21.17 keV, Lα1 2.8 keV), or artefacts found in some pigments at 4.4 keV or at 9.2 keV due to sum or escape peaks. Rh, Ni, and Pd are referred to internal instrumental components of the Tracer III SD AXS portable spectrometer, as well the signal of argon is due to the environmental atmosphere contribute. For the statistical analysis, the lines Kα are considered for the elements Mg, Al, Si, P, S, K, Ca, Ti, Cr, Mn, Fe, Co, Cu, Zn, As, Sr, Sn, Sb; for the heavy metals Hg and Pb the following lines are considered HgLα1 and PbLα1. The MnKα peak is fundamental for the identification of the Schist Earth pigment [23]. No Compton normalization is carried out because for some pigments is negligible (for those characterised by heavy-metals) except for the real-case sample where the Ca/Sr and the other elements are attributed to the deterioration processes (carbonatation and phosphating) and constituted the main peaks. The counts were obtained by selecting the net counts of the Kα line of most of the elements detected and the Lα1 line for Hg and Pb. Baseline subtraction is applied to the entire raw dataset [24]. 

**Raman and IR data treatment**. In view of building the matrix and comparing the data with each other an accurate data treatment is carried out. Firstly, a linear interpolation method is applied to the entire dataset obtaining the same energy step, of 2 cm^−1^, on the x-axis. For the Vertex 70 instrument the spectra are cut off at 3999 cm^−1^ once verified the absence of significant peaks. In this way, the assigned variables are 1999 for all the matrices where Raman and FT-IR are considered. Finally, the intensities of the Raman and FT-IR spectra are normalized between 0–1 [23]. For each one technique, a total of 316 measurements are recorded. A selection of spectra is then made keeping out the undetectable or noise signals obtaining a total of 220 spectra. For the XRaman the following samples are considered into the data matrix RH, RL, RCR, RM, WL, WSG, WB, YG, YN, YOR, BLA, BLL, EV (the correspondence between the labels and the pigments in Figure 1 are reported in Supplementary, Table S1). The greens are kept out as well as the black ones, the lake pigment, and the organic pigments. Regarding the BRAVO dataset, the following pigments are not considered: RCE, GC, GJ, GM, BY, BV, RD, BLS, and GV, BG, RCR, and BL are eliminated from the Vertex 70 data matrix because no significant signals are visible.

### 2.7. Matrix Building and PCA Data Processing

Three data set matrices are built with two incomes: one for the samples’ measurements, one for the elements and the latter for the Energy (column data). They are imported into the Unscrambler X software (CamoAnalytics) and treated by PCA. Due to the different sizes of FT-IR and Raman data outgoing a unique Energy range from 2 to 3999 cm^−1^ is used. To facilitate the interpretation of the results, three different PCA are shown, one for XRF data, one for the inorganic pigments and one for the organic ones in which all the other two techniques (Raman and FT-IR) are considered. In this sense, 220 measurements (between the FTIR/Raman inorganic and organic matrices) are considered in the PCA analysis. SVD (XRF and FTIR/Raman organic matrices) and NIPALS (FTIR/Raman inorganic matrix) algorithms are used with 100 as the value of iterations (the data are not noisy and 100 are sufficient to converge the data). As a validation method, a Cross-validation (the number of the sample is moderate) and a Random with 20 segments are chosen. To obtain the maximum convergence, on all the variables a Standard Deviation (SDev) is applied as standardization weight on the Raman and FT-IR spectra (inorganic and organic matrices). A total of two optimal numbers of components explained a total of variance for each PCA. The optimal number of components corresponds with the number of independent phenomena in the data that exceeds the noise level of the measurements. In detail, the three built matrices are the following: (a) 1 matrix with 97 × 20 size for the XRF technique (XRaman, Tracer III), (b) 1 matrix with 81 × 1999 size for the Raman and FT-IR techniques where organic pigments are considered, and (c) 1 matrix with 139 × 1999 size for the Raman and FT-IR techniques to represent the inorganic pigments. 

## 3. Results and Discussion

**Figure 3:**
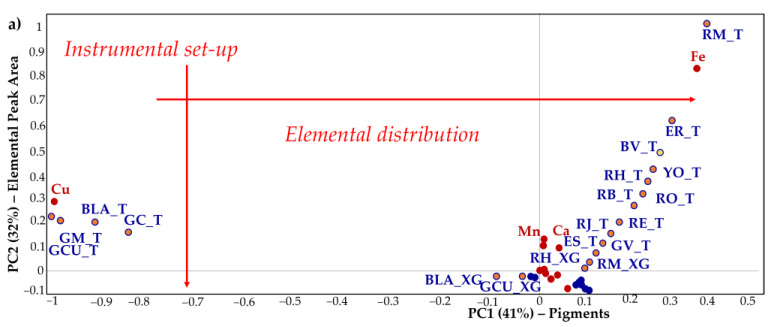

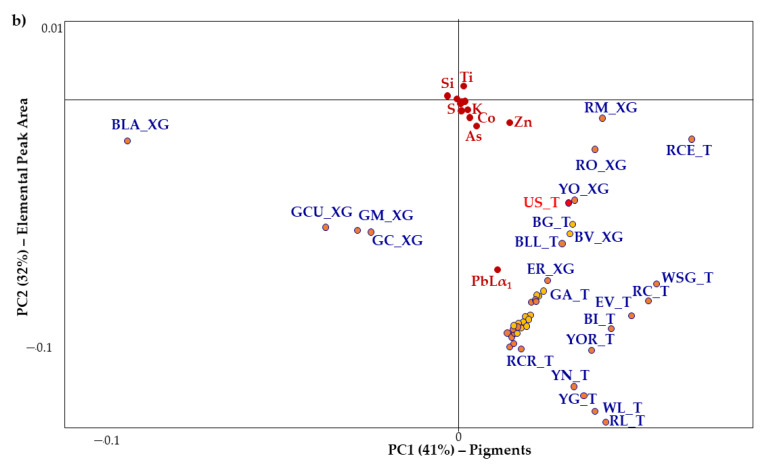
XRF Bi-Plot (loading plot in red colour and score plot in blue). (**a**) General Bi-plot overview; (**b**) zoom of the III and IV quadrants. The sample points are coloured by orange and yellow if they are inorganic or organic respectively. US_T sample is the real-case sample where the elemental constituents are unknown.

**XRF spectroscopy**. Figure 3 shows the Bi-plot, a two-dimensional scatter plot or map of scores used to interpret samples’ properties (Scores plot, point measurements distribution or Elemental Peak Area in this case), with the variables’ properties (X-Loadings, elemental distribution) displayed on the same plot. In this way, sample and variable relationships are interpreted simultaneously. The distributional correlation analysis of the XRF measurements (samples) with the corresponding elements (variables) is observed. In detail, two principal components with 41% and 32% (PC1 and PC2 respectively) explain the 73% of the total variance. The PCA (Figure 3a) shows two main groups, characterised by Cu and Fe, founded in opposites (II and I quadrant respectively) because in our measurements these elements are not found in the same pigment. The first group includes the Cu-based inorganic pigments (blue-green colour) while the second group includes the Fe-based inorganic and organic pigments (reddish-yellow-brown ochre). Along the PC1 that is associated to the elemental distribution, the samples are distributed according to their colour scale: in the Cu group, for example, the Verdigris (GCU), Malachite (GM), Azurite (BLA), and Chrysocolla (GC) are distributed from the green to the blue/green colours; a similar trend is observed for the red/yellow ochre that spans from the dark reddish-brown (RM) to the yellow (YG, IV quadrant). The association with the colour and its shadows is linked with the predominant elemental marker/chromophore that is the element with the higher peak area. Along the PC1, the amount of the predominant marker increases from 0 to 0.5 variance, which corresponds to the *Morellone* (RM, I quadrant) where the high correlation with Fe shows the purity of this pigment. The Fe element is the discriminant element in the red ochre and ochres in general and how it is gradually close to positive PC1 values, its amount decreases and new other elements taking place (e.g., Ca, Zn, Ti, Mn, etc.). This is the case of the red and yellow-green earths where the Ca has a not-negligible weight, linked with the carbonate mother rock. Along the PC2, the data are distributed according to the instrumental setup. Looking at lower variances (Figure 3b) other elements such as Ca, Zn, Pb, As, Co, Ti etc. can be considered as markers of the dataset. 

As the variance moves at values near to zero the pigments are characterized by more than one element, reducing the contribution of the predominant marker. In this way, the minor elements are markers of their mineralogical-geological origin such as the K and Sr or Ti (terrigenous input) and accessory phases including but not limited to clays, carbonates, and quartz. In this sense, the pigments show a more complex fingerprint (geological and mineralogical features). This is the case of K, a crucial element of phyllosilicates like micas, illite, or smectites which are essential minerals in schist or the Ti, an element usually associated with the terrigenous component of the sediments, that could be more or less important in each ochre. In this context, besides Cu or Fe, the pigments are enriched in metals like Pb, As, and Zn, which can be used as fingerprints [24,25]. The PC2 highlights the different set-up of the ED-XRF instruments in which the same pigments are detected in terms of intensity counts differently. 

Considering the real-case, its values fall along the line where the Fe is the elemental benchmark according with the assignment of red earth and Fe-based pigments in the paper of the study [22] and indicating that the statistical approach allows the automatic identification of the benchmark and a possible assignment to a pigment‘s group (i.e., red ochres). Despite the measure of the real case also containing signals coming from a complex matrix (pigment, binder, preparation) the interesting result highlights the ability of the proposed approach for the automatic identification of benchmarks making the interpretation of the spectra faster and simpler. 

**Figure 4:**
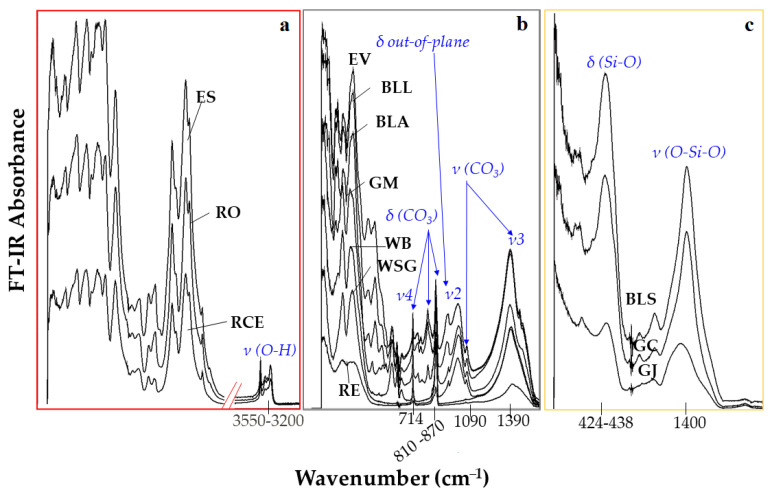
FTIR spectra analysed by Vertex 70 instrument: iron oxides (**a**), carbonates (**b**) and silicates (**c**).

**FT-IR spectroscopy.** Figure 4 shows the FT-IR spectra pattern for iron oxides (a), carbonates (b) and silicates (c), analysed by Vertex 70 instrument. The first group has the same chromophore, iron (II) oxide (FeO), which its characteristic spectrum (Figure 4a). The second group is constituted by carbonates (WB, WSG, BLL, GM, RE), even if red ochre is mainly constituted by iron oxide, the appearance of calcite peaks in FTIR spectra indicates the presence of this compound as an impurity as BLA and/or white earth (EV). Regarding BLL, most lapis lazuli also contain calcite (white), together with sodalite (blue), and pyrite (metallic yellow) [26]. All these pigments have in common the bending and stretching of the ion (CO_3_)^2-^ (Figure 4b). In detail, calcite-group carbonates include the major absorption bands at 1400 cm^−1^ and between 700 cm^−1^ and 200 cm^−1^ each of which may be identified with a particular deformational mode, ν2-ν4, of the (CO_3_)^2−^ ion [27,28]. In the third group, the silicates spectra (GJ, GC, BLS) show a broad band around 1000 cm^−1^ related to the Si–O–Si stretching, the fingerprints region responsible for their discrimination [27].

**Figure 5:**
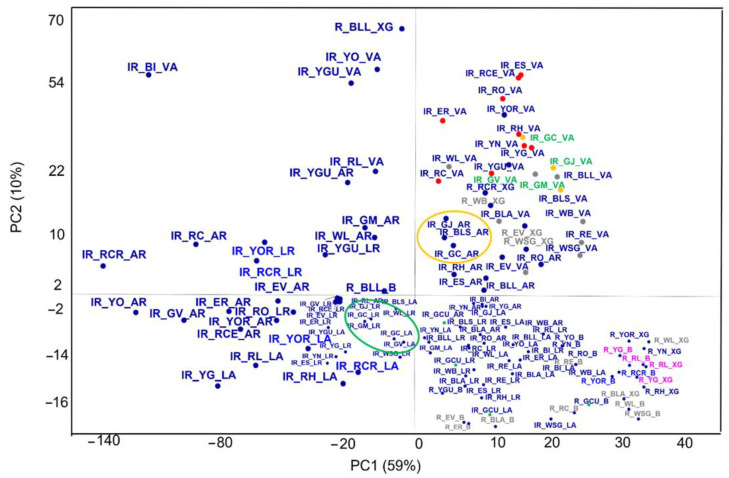
Scores plot (point measurements distribution+) of the Raman and FT-IR measurements for the inorganic pigments. The iron-oxides are indicated by red points, the silicates are indicated by yellow points, the green pigments are highlighted by green labels and green circle; the carbonates are reported with grey labels; the orpiment and cinnabar (YOR and RCR) are labelled in blue; the red lead and *Giallorino* (RL and YG) are reported in lilac.

**Figure 6:**
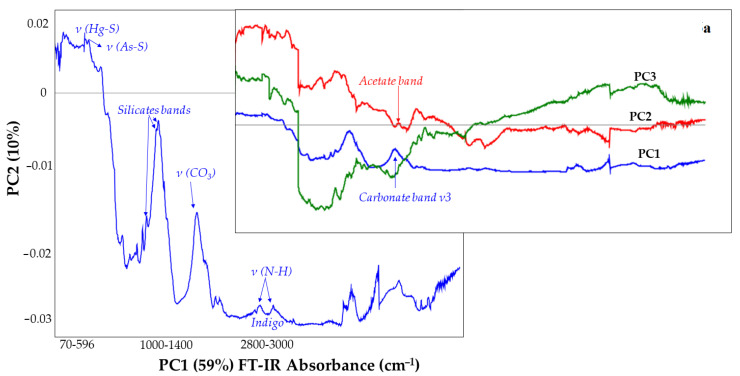
Loadings plot: correlation of the FT-IR Absorbance frequency (cm^−1^) of the entire spectral ranges for the Raman and FT-IR measurements applied to the inorganic pigments’ matrix. The inset 6a shows the three PCs (PC1–blue line, PC2–red line and PC3–green line) as function of the Energy. The spectroscopic fingerprints of the molecular motion involved on the entire FTIR dataset are shown in each PC. The PC1 explains 59% of the most significant vibrational modes.

**Figure 7:**
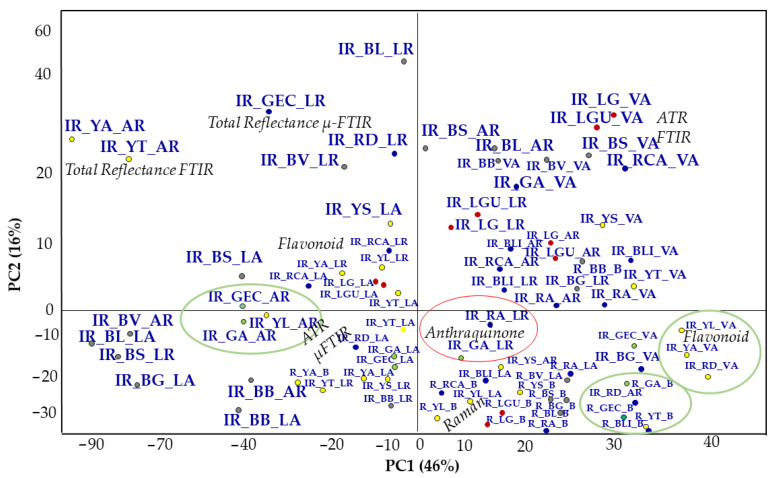
Scores plot (measurements distribution) of the Raman and FT-IR spectra for the organic pigments. The flavonoids are indicated by yellow and green points (wood), and they are grouped by a green circle; the lake pigment are indicated by red points; the anthraquinones are signed by red points and grouped by red circles. The black pigments acquired by LUMOS in ATR are separated along PC2.

**Figure 8:**
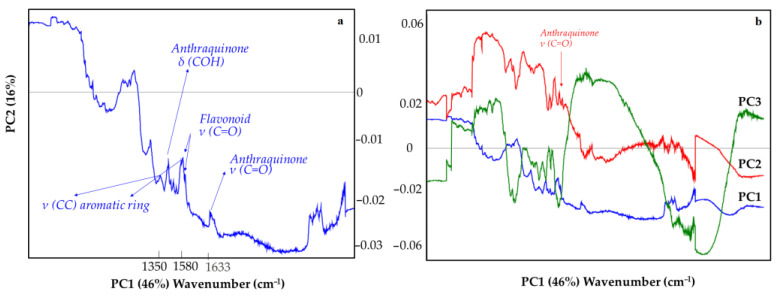
Loadings plot (variable distribution projection, wavenumber cm^−1^) in line plot for the PC1 visualization (to highlight the spectral regions generated by the first component) where some of the vibrational molecular modes for the organic pigments are shown. The variables are normalized at step 2 cm^−1^ on the entire energy spectrum (see (**b**) the Raman and (**a**) IR data treatment section).

**Raman and IR spectroscopies.** Figure 5, Figure 6, Figure 7 and Figure 8 show the Score plots and the Loadings plots related to the inorganic and organic pigments, respectively. Starting from the inorganic pigments (Figure 5), the Scores plot shows three PCs with a total of the explained variance of 75%. PC1 that carries the major contribution (59%) well separated the entire dataset according to the instrument of belonging: the PCA discriminates each technique classifying it in a distinct group. Indeed, the instruments are well grouped according to their discriminate properties. In general, the FT-IR data present distribution values greater thanks to their wide range of measure than the Raman ones which are closer together in the lower right-hand corner where PC1 is positive whilst the PC2 is negative (except the EV and WSG and WB analysed by XRaman, set both in the I quadrant, highlighted in Figure 5 in grey colour—where the peak at 1086 cm^−1^ of the calcites mainly stands out). For each one instrument, it is possible to observe some groups according to the class of belonging. Starting by the I quadrant, Vertex 70 spectrometer (ATR mode) grouped a wide pigments cluster of different origin: (a) one group of earths characterized by oxides, iron and lead or iron carbonate (YN, YG, GV, ER, RCR, RM, WL, etc.) with characteristic bands in the wavenumber region below 700 cm^−1^ [26] and iron oxides (ES, RCE, RO) reported in Figure 5 as red points; (b) a carbonates’ groups (reported in Figure 5 as grey points), WB, WSG, BLL, GM, RE, according to the high calcium content identified by XRF analysis, BLA (reported in Figure 5 as grey points) or white earth (EV); (c) a silicates group (GJ, GC, BLS reported in Figure 5 as orange points); (d) a subgroup of green colours’ pigments (GV, GM, GJ, and GC—Figure 5, green labels).

The Alpha (R mode) spreads along the PC2 (I-I, II, III, and IV quadrants). It is possible to distinguish: a silicates group (GJ, BLS, and GC–Figure 5, orange circle), some carbonate groups (BLL, EV–GM, WL), and iron oxides (RH, ES). Looking at the IV quadrant it is possible to observe the Raman measurements that well grouped the lead oxides (RL and YG in Figure 5, lilac label), carbonates (WL, WSG, BLA, RC in Figure 5, grey label) or earth characterized by CO_3_^2-^ spectroscopic features (EV, ER) and those characterized by C-S vibration modes at 344 cm^−1^ [27] (YOR and RCR in Figure 5, blue label). Indeed, looking at the Loadings Plot (Figure 6) the fingerprint of each class of pigments marks the data distribution. The As-S stretching at 355 cm^−1^ (and S-As-S bending regions are identified for orpiment) [29] and Hg-S stretching (characteristic of the cinnabar) are spectroscopic benchmarks (each one instrument highlights this: YOR and RCR are always set very close to each other—Figure 5, blue label) as well as N-H stretching (Figure 6), vibrational mode of the indigo pigment. 

To highlight the spectroscopic features of the whole data set, three Loadings plot for each one PC (Figure 6) are carried out (in line plot mode) to individuate those spectroscopic benchmarks characteristics of the pigments under study. Indeed, it is possible to observe how some vibrations are more significant than others. This is the case of the compound as the GCU where the acetate band, around 1400 cm^−1^ falls in the same spectral region of the carbonate one (ν3–see PC1 blue line and PC2 red line–Figure 6). Moreover, in the Scores plot reported in Figure 5 the Verdigris pigment (Figure 5–green points) is always positioned close to one or more carbonate (in all three cases: BRAVO, LUMOS in Reflection and ATR mode). Moreover, the acetate band is distinguished by the PC2: the GCU is the single copper acetate present in the whole data set. Other interesting vibrations are those where the silicate bands are involved: 1159 cm^−1^, 1042 cm^−1^, 1000 cm^−1^ (PC1–Figure 6) [30,31,32,33]. In Figure 5, the LUMOS instrument well grouped the green pigments (Figure 5, green circle). The Alpha and the LUMOS, both in Reflection mode, fall close and clustered the iron oxides as well in the LUMOS in ATR mode. Figure 7 indicates the PCA result from the organic pigments. The total explained variance is 72% (49% PC1, 16% PC2 and 10% PC3). At first sight, it is possible to observe that each technique well separates pigments according to their molecular benchmark. Each technique discriminates against the lake pigment (Figure 7—red points), anthraquinones (Figure 7—red circle), and the flavonoids (Figure 7—green circle). The discrimination works both for Vertex and Lumus instruments as demonstrated by the presence of well-defined groups for each instrument.

Among the groups by blacks’ colour, the carbon black based pigments show characteristic bands at approximately 1580 cm^−1^ (aromatic ring CC stretching vibration) [11] and 1350 cm^−1^ [11]. The data set contains mainly vegetal pigments, above all flavonoids, plants, leaves, or flowers well grouped by the Vertex 70 spectrometer (Figure 7—green circle) and Alpha one, where some pigments are extracted by; indeed, they are a large class of polyphenols widely distributed in plants and so in pigments. The characteristic wavelength regions are two: 5840–6090 cm^−1^ and 6620–6880 cm^−1^, which correspond to the absorptions of two aromatic rings in a basic flavonoid structure. Here, it is possible to observe the vibration at 1709 cm^−1^ belongs to the -C=O stretching of flavonoid. This group (-C=O) conjugates with the ring double bonds giving a -C=C- stretching vibration at 1631–1611 cm^−1^ (Figure 8a). Amongst the FT-IR spectrometers, no differences are detected between the two operational modes (total or attenuated reflection). The benchmarks for this compound are characterized by the aromatic and carbonyl group in the flavonoid structure that operates as a conjugated chromophore. Regarding the anthraquinones (LG, RA, GA, and RCA) their colours depend on the position and number of hydroxyl substituents [34]: these dyes have O–H and C=O stretching vibrations modes at 3624–3544 cm^−1^ and 1685–1674 cm^−1^, respectively, that are strong vibration modes.

## 4. Conclusions

A systematic study of 48 natural powdered pigments is carried out via spectroscopic analyses together with Principal Component Analysis and tested on a real archaeological sample. This methodology allows access to the unambiguous elemental and molecular benchmarks used for the identification of an unknown pigment and its recipe. Benchmarks/chromophores, and spectral fingerprint in the pigment identification are obtained on the entire dataset acquired through four spectroscopic techniques (X-ray Fluorescence, Raman, Attenuated Total Reflectance, and Total Reflectance Infrared Spectroscopies) by different instrumental devices and setup.

XRF-PCA has highlighted two different trends headed by two main benchmarks/chromophores: Cu-based inorganic pigments (blue-green colour) while the second group includes the Fe-based inorganic and organic pigments (reddish-yellow-brown ochre). The colour and shadows are effectively linked with the main element and with other markers that gradually take place associated with carbonate mother rock, Ca, and the geological and mineralogical features, as K and Sr or Ti. In this sense, it was possible to associate each group of categories (red-ochre earth) and thus to each colour (red yellows) to the main benchmarks/chromophores. The test on the real case demonstrates the potentiality of the separation technique in which the sample added to the data matrix falls in the red-yellow ochre. This means that unknown samples or where the recipes can change (from some minor elements linked to the raw materials, geological context) can be easily classified following the main benchmark/chromophore, also in the presence of degradation products. In the same way, spectroscopic benchmarks are found in FT-IR spectroscopy. Iron and lead or iron carbonate, bending and stretching of the ion (CO_3_)^2−^, and Si–O–Si stretching, are the vibrations markers for iron oxides (red-yellow earths), carbonates (green, red, and white earths), and silicates (green-blue) respectively. Additional stretching like N-H and Hg-S are distinct vibrations modes and spectral benchmarks for specific pigments like cinnabar and indigo, radical chromophores of a specific class of pigments (such as azo dyes).

In conclusion, the approach demonstrates that it is possible to classify specific categories amongst inorganic and organic pigments by the identification of elemental and molecular benchmarks using statistical analysis to identify trends on even non-homogeneous data sets. Future perspectives are opened by the implementation of the matrix by new input data, such as the pigments mixture, binders and recipes demonstrating that although the system becomes more complex, it will be possible to identify their benchmarks.

## Data Availability

The datasets generated during and/or analysed during the current study are available from the corresponding author on reasonable request.

## Supplementary Materials

The following are available online, Table S1: List of pigments. The table reports the category of belonging, the name of the pigment, the class of belonging (Organic or Inorganic) and the ID labels assigned; Table S2: XRF matrix. On the columns, the element detected and selected, on the rows the XRF the measurements for each one pigments. The normalized peak areas have a ±0.01 error associated. US_T stands for Unknown samples, tested with the TRACER III.

## Sample Availability

Samples of the compounds are purchasable from the Zenchi company.
