# Supplementary material for: Chemometric Tools to Point Out Benchmarks and Chromophores in Pigments through Spectroscopic Data Analyses
Source: Molecules. 2021 Dec 28;27(1):163. doi: 10.3390/molecules27010163 (PMC8746391; doi:10.3390/molecules27010163)
Supplement: Supplementary file 1 [file molecules-27-00163-s001.zip › molecules-1473770-supplementary.pdf]

Chemometric Tools to Point Out Benchmarks and Chromophores in Pigments through Spectroscopic Data Analyses

Giulia Festa[1], Claudia Scatigno*[1], Francesco Armetta*[2], Maria Luisa Saladino[2], Veronica Ciaramitaro[2], Viviana Mollica Nardo[3] and Rosina Celeste Ponterio[3]

[1] CREF - Museo Storico della Fisica e Centro Studi e Ricerche "Enrico Fermi", Via Panisperna 89 a, c/o Piazza del Viminale 1, 00189, Roma, Italy; giulia.festa@cref.it, claudia.scatigno@cref.it

[2] Dipartimento Scienze e Tecnologie Biologiche, Chimiche e Farmaceutiche -STEBICEF and INSTM UdR -Palermo, Università di Palermo, Viale delle Scienze Bld.17, Palermo I-90128, Italy; francesco.armetta01@unipa.it, marialuisa.saladino@unipa.it, ciaramitaroveronica@gmail.com

[3] CNR - Istituto per i Processi Chimico Fisici Laboratorio di Tecniche Spettroscopiche (LATES), Viale Ferdinando Stagno d'Alcontres, 37, Messina I -98158, Italy; mollica@ipcf.cnr.it, ponterio@ipcf.cnr.it

* Corresponding author:
C.S.claudia.scatigno@cref.it; F.A. francesco.armetta01@unipa.it

Table of Contents

Table S1. List of pigments……..………………………………………………………………………….S3
Table S2. XRF matrix. ………………………………………………………………………………S4

For detailed description of the analyzed pigments please see the Primary Research Article.

Table S1. List of pigments. The table reports the category of belonging, the name of the pigment, the class of belonging (Organic or Inorganic) and the ID labels assigned.

Category	Name	Org/Ino	ID
**Red**	Hematite	Ino	RH
	Ercolano	Ino	RE
	Cinabrese	Ino	RCE
	Carmine	Org	RCA
	Red Ochre	Ino	RO
	Dragon's blood	Org	RD
	Coral	Ino	RC
	Red Lead	Ino	RL
	Alizarin	Org	RA
	Cinnabar	Ino	RCR
	Bolo	Ino	RB
	Jasper	Ino	RJ
	Morellone	Ino	RM
**White**	White Lead	Ino	WL
	White *San Giovanni*	Ino	WSG
	White Bone	Ino	WB
**Green**	Malachite	Ino	GM
	Campeggio	Org	GEC
	Chrysocolla	Ino	GC
	Aloe	Org	GA
	Brazilwood	Org	GB
	Jasper	Ino	GJ
	Verdaccio	Ino	GV
	Verdigris	Ino	GCU
**Yellow**	Arzica	Org	YA
	Saffron	Org	YS
	Turmeric	Org	YT
	Yellow Ochre	Ino	YO
	Giallorino	Ino	YG
	Yellow Naple	Ino	YN
	Gommagutta	Org	YGU
	Orpiment	Ino	YOR
	Stil de grain	Org	YL
**Blue**	Smalt	Ino	BLS
	Azurite	Ino	BLA
	Indigo	Org	BLI
	Lapis lazuli	Ino	BLL
**Black**	Black Lamp	Org	BL
	Ivory	Ino	BI
	Sepia	Org	BS
	Bitumen	Org	BB
	Vine Black	Org	BV
	Graphite	Org	BG
**Earth**	Vicenza Earth	Ino	EV
	Romana Earth	Ino	ER
	Schist	Ino	ES
**Lacquer**	*Gommalacca*	Org	LGU
	Garanza	Org	LG

* For each one new ID that will be used for the PCA analysis will be assign a code: the initial capital letter stands for the technique used (XRF, R=Raman, IR=FT-IR); the next capital letter indicates the class of pigments' membership, R for red, W for white, G for green, Y for yellow, BL for blue, B for black, E for earths and L for lacquers. Finally, the initial name of the pigment and the last one refers to the instrument used (T for TRACER III, XG for XRaman, B for BRAVO, AR for alpha in reflection mode, VA for Vertex 70 in attenuated reflection mode, LA for LUMOS in attenuated reflection mode and LR for LUMOS in total reflection mode). Each information is separated from the successive by the underscore.

Table S2. XRF matrix. On the columns, the element detected and selected, on the rows the XRF the measurements for each one pigments. The normalized peak areas have a ±0.01 error associated. US_T stands for Unknown samples, tested with the TRACER III.

	Mg	Al	Si	P	S	K	Ca	Ti	Cr	Mn	Fe	Co	Cu	Zn	As	Sr	Sn	Sb	$HgL_{\alpha1}$	$PbL_{\alpha1}$
BLA_T	nd	nd	nd	nd	nd	nd	nd	nd	nd	nd	nd	nd	41379	nd	nd	nd	nd	nd	nd	nd
BLA_XG	nd	nd	nd	nd	nd	nd	nd	nd	nd	nd	nd	nd	5248	nd	nd	nd	nd	nd	nd	nd
BU_T	nd	nd	nd	84	39	350	1644	55	nd	125	432	nd	35	149	nd	nd	nd	nd	nd	nd
BU_XG	nd	nd	nd	4	2	26	132	6	nd	12	36	nd	3	26	nd	nd	nd	nd	nd	nd
BLL_T	nd	nd	92	nd	111	180	1014	80	nd	nd	921	nd	nd	nd	nd	nd	nd	nd	nd	nd
BLL_XG	nd	nd	5	nd	5	20	96	9	nd	nd	84	nd	nd	nd	nd	nd	nd	nd	nd	nd
BLS_T	nd	nd	444	nd	nd	1608	nd	nd	nd	nd	nd	11146	nd	nd	nd	nd	nd	nd	nd	nd
BLS_XG	nd	nd	14	nd	nd	80	nd	nd	nd	nd	nd	554	nd	nd	nd	nd	nd	nd	nd	nd
WL_T	nd	nd	nd	nd	nd	nd	nd	nd	nd	nd	nd	nd	nd	nd	nd	nd	nd	nd	nd	19922
WL_XG	nd	nd	nd	nd	nd	nd	nd	nd	nd	nd	nd	nd	nd	nd	nd	nd	nd	nd	nd	1033
WB_T	nd	nd	nd	618	nd	nd	32	nd	nd	nd	nd	nd	nd	nd	nd	nd	nd	nd	nd	nd
WB_XG	nd	nd	nd	8831	nd	nd	599	nd	nd	nd	nd	nd	nd	nd	nd	nd	nd	nd	nd	nd
WSG_T	nd	nd	nd	nd	nd	nd	14517	nd	nd	nd	nd	nd	nd	nd	nd	nd	nd	nd	nd	nd
WSG_XG	nd	nd	nd	nd	nd	nd	1015	nd	nd	nd	nd	nd	nd	nd	nd	nd	nd	nd	nd	nd
BB_T	nd	nd	nd	nd	nd	nd	nd	nd	nd	nd	199	nd	nd	nd	nd	nd	nd	nd	nd	nd
BB_XG	nd	nd	nd	nd	3	nd	1	nd	nd	nd	10	nd	nd	nd	nd	nd	nd	nd	nd	nd
BG_T	nd	nd	42	5	nd	212	1140	187	nd	52	1138	nd	nd	nd	nd	nd	nd	nd	nd	nd
BG_XG	nd	nd	2	1	nd	13	66	10	nd	4	47	nd	nd	nd	nd	nd	nd	nd	nd	nd
BI_T	nd	nd	nd	584	nd	nd	702	nd	nd	nd	nd	nd	nd	nd	nd	nd	nd	nd	nd	nd
BI_XG	nd	nd	nd	19	nd	nd	338	nd	nd	nd	nd	nd	nd	nd	nd	nd	nd	nd	nd	nd
BV_T	nd	nd	13	nd	3	nd	1460	459	nd	2982	23346	nd	nd	nd	nd	nd	nd	nd	nd	nd
BV_XG	nd	nd	nd	nd	nd	nd	77	nd	nd	129	1022	nd	nd	nd	nd	nd	nd	nd	nd	nd
BS_T	nd	nd	nd	nd	50	105	788	nd	nd	nd	262	nd	nd	nd	nd	nd	nd	nd	nd	nd
BS_XG	nd	nd	nd	nd	3	6	31	nd	nd	nd	7	nd	nd	nd	nd	nd	nd	nd	nd	nd
BL_T	nd	nd	nd	nd	nd	nd	157	nd	nd	nd	nd	nd	nd	nd	nd	nd	nd	nd	nd	nd
BL_XG	nd	nd	nd	nd	nd	nd	3	nd	nd	nd	nd	nd	nd	nd	nd	nd	nd	nd	nd	nd
LG_T	nd	nd	nd	nd	nd	nd	27	19	nd	nd	73	nd	nd	nd	nd	nd	nd	nd	nd	nd
LG_XG	nd	nd	nd	nd	nd	nd	11	3	nd	nd	4	nd	nd	nd	nd	nd	nd	nd	nd	nd
LGU_T	nd	nd	nd	6	nd	nd	38	26	nd	nd	72	nd	nd	nd	nd	nd	nd	nd	nd	nd
LGU_XG	nd	nd	nd	nd	1	3	4	nd	nd	nd	5	nd	nd	nd	nd	nd	nd	nd	nd	nd
ES_T	nd	16	174	nd	nd	378	110	227	nd	nd	5747	nd	nd	nd	nd	nd	nd	nd	nd	nd
ES_XG	nd	1	12	nd	nd	37	12	18	nd	nd	419	nd	nd	nd	nd	nd	nd	nd	nd	nd
US_T	nd	nd	17	45	2	52	13	0,1		0	1532	nd	nd	nd	nd	13	nd	nd	nd	nd
EV_T	nd	nd	nd	nd	nd	19	10585	238	nd	nd	nd	nd	nd	nd	nd	nd	nd	nd	nd	nd
EV_XG	nd	nd	nd	5	nd	3	587	7	nd	nd	nd	nd	nd	nd	nd	nd	nd	nd	nd	nd
ER_T	nd	nd	171	35	nd	nd	9278	1165	nd	421	29385	nd	nd	nd	nd	nd	nd	nd	nd	nd
ER_XG	nd	nd	4	nd	nd	nd	217	21	nd	9	574	nd	nd	nd	nd	nd	nd	nd	nd	nd
YT_T	nd	nd	nd	19	30	1564	nd	43	nd	nd	346	nd	nd	nd	nd	nd	nd	nd	nd	nd
YT_XG	nd	2	nd	2	1	97	nd	1	nd	nd	15	nd	nd	nd	nd	nd	nd	nd	nd	nd
YS_T	nd	nd	nd	26	35	976	234	27	nd	nd	224	nd	61	37	nd	nd	nd	nd	nd	nd
YS_XG	nd	nd	nd	21	30	976	234	27	nd	nd	230	nd	54	25	nd	nd	nd	nd	nd	nd
YA_T	nd	39	nd	nd	595	939	137	27	nd	nd	60	nd	nd	nd	nd	nd	nd	nd	nd	nd
YA_XG	1	4	nd	nd	47	104	nd	nd	nd	nd	nd	nd	nd	nd	nd	nd	nd	nd	nd	nd
YN_T	nd	nd	nd	nd	698	nd	nd	nd	nd	nd	nd	nd	nd	nd	nd	nd	nd	316	nd	10748
YN_XG	nd	nd	nd	nd	6	nd	nd	nd	nd	nd	nd	nd	nd	nd	nd	nd	nd	168	nd	1510
YG_T	nd	nd	nd	nd	nd	nd	nd	nd	nd	nd	nd	nd	nd	nd	nd	nd	531	nd	nd	14902
YG_XG	nd	nd	nd	nd	nd	nd	nd	nd	nd	nd	nd	nd	nd	nd	nd	nd	64	nd	nd	1043
YO_T	nd	nd	18	nd	nd	nd	376	nd	nd	nd	22401	nd	nd	nd	nd	nd	nd	nd	nd	nd
YO_XG	nd	nd	1	nd	nd	nd	30	nd	nd	nd	1533	nd	nd	nd	nd	nd	nd	nd	nd	nd
YGU_T	nd	nd	nd	nd	nd	15	35	nd	nd	nd	nd	nd	nd	nd	nd	nd	nd	nd	nd	nd
YGU_XG	nd	nd	nd	nd	nd	6	9	nd	nd	nd	nd	nd	nd	nd	nd	nd	nd	nd	nd	nd
YL_T	nd	64	nd	nd	1103	1012	123	nd	nd	nd	nd	nd	nd	nd	nd	nd	nd	nd	nd	nd
YL_XG	nd	3	nd	nd	52	59	nd	nd	nd	nd	nd	nd	nd	nd	nd	nd	nd	nd	nd	nd
YOR_T	nd	nd	nd	nd	1550	nd	nd	nd	nd	nd	288	nd	nd	nd	24176	nd	nd	nd	nd	nd
YOR_XG	nd	nd	nd	nd	86	nd	nd	nd	nd	nd	10	nd	nd	nd	903	nd	nd	nd	nd	nd
RA_T	nd	nd	nd	nd	9	nd	59	25	nd	nd	386	nd	nd	nd	nd	nd	nd	nd	nd	nd
RA_XG	nd	nd	nd	nd	1	nd	4	3	nd	nd	28	nd	nd	nd	nd	nd	nd	nd	nd	nd
RD_T	nd	nd	nd	nd	14	nd	105	33	9	nd	161	nd	nd	nd	nd	nd	nd	nd	nd	nd
RD_XG	nd	nd	nd	nd	nd	nd	10	nd	nd	nd	12	nd	nd	nd	nd	nd	nd	nd	nd	nd
RL_T	nd	nd	nd	nd	273	nd	nd	nd	nd	nd	nd	nd	nd	nd	nd	nd	nd	nd	nd	24028
RL_XG	nd	nd	nd	nd	71	nd	nd	nd	nd	nd	nd	nd	nd	nd	nd	nd	nd	nd	nd	861
RB_T	nd	nd	225	nd	nd	277	nd	494	nd	nd	17152	nd	nd	nd	nd	nd	nd	nd	nd	nd
RB_XG	nd	nd	6	nd	nd	14	nd	17	nd	nd	489	nd	nd	nd	nd	nd	nd	nd	nd	nd
RM_T	nd	nd	nd	nd	nd	nd	nd	nd	nd	nd	54804	nd	nd	nd	nd	nd	nd	nd	nd	nd
RM_XG	nd	nd	nd	nd	nd	nd	nd	nd	nd	nd	2894	nd	nd	nd	nd	nd	nd	nd	nd	nd
RC_T	nd	nd	nd	99	nd	nd	12971	nd	nd	nd	6	nd	nd	nd	nd	nd	nd	nd	nd	nd
RC_XG	nd	nd	nd	3	nd	nd	235	nd	nd	nd	nd	nd	nd	nd	nd	nd	nd	nd	nd	nd
RCA_T	nd	nd	nd	26	61	27	1851	25	nd	nd	198	nd	67	nd	nd	nd	nd	nd	nd	nd
RCA_XG	nd	nd	nd	2	4	4	105	3	nd	nd	8	nd	3	12	nd	nd	nd	nd	nd	nd
RE_T	nd	nd	nd	nd	1293	nd	5254	nd	nd	nd	9033	nd	nd	nd	nd	nd	nd	nd	nd	nd
RE_XG	nd	nd	nd	nd	52	nd	291	nd	nd	nd	412	nd	nd	nd	nd	nd	nd	nd	nd	nd
RH_T	nd	nd	nd	nd	nd	nd	nd	nd	nd	nd	21844	nd	nd	nd	nd	nd	nd	nd	nd	nd
RH_XG	nd	nd	nd	nd	nd	nd	nd	nd	nd	nd	3760	nd	nd	nd	nd	nd	nd	nd	nd	nd
RCR_T	nd	nd	nd	nd	1018	nd	nd	nd	nd	nd	nd	nd	nd	542	nd	nd	nd	nd	13313	nd
RCR_XG	nd	nd	nd	nd	88	nd	nd	nd	nd	nd	nd	nd	nd	41	nd	nd	nd	nd	955	nd
RJ_T	nd	nd	596	nd	nd	nd	201	nd	nd	nd	8123	nd	nd	nd	nd	nd	nd	nd	nd	nd
RJ_XG	nd	nd	12	nd	nd	nd	8	nd	nd	nd	221	nd	nd	nd	nd	nd	nd	nd	nd	nd
RO_B	nd	nd	nd	nd	nd	nd	nd	nd	nd	nd	18358	nd	nd	nd	nd	nd	nd	nd	nd	nd
RO_XG	nd	nd	nd	nd	nd	nd	nd	nd	nd	nd	2050	nd	nd	nd	nd	nd	nd	nd	nd	nd
RCE_B	nd	nd	nd	nd	nd	7	nd	nd	nd	nd	2437	nd	nd	41868	nd	nd	nd	nd	nd	nd
RCE_XG	nd	nd	nd	nd	nd	14	nd	nd	nd	nd	102	nd	nd	2316	nd	nd	nd	nd	nd	nd
GC_T	nd	nd	13	nd	nd	nd	nd	nd	nd	346	409	nd	36951	nd	nd	nd	nd	nd	nd	nd
GC_XG	nd	nd	4	nd	nd	nd	nd	nd	nd	15	24	nd	1775	nd	nd	nd	nd	nd	nd	nd
GJ_T	nd	nd	98	nd	669	nd	410	nd	nd	nd	18	nd	nd	nd	nd	nd	nd	nd	nd	nd
GJ_XG	nd	nd	2	nd	22	nd	16	nd	nd	nd	3	nd	nd	nd	nd	nd	nd	nd	nd	nd
GV_T	nd	nd	nd	nd	1073	nd	5552	nd	nd	8847	7224	nd	nd	nd	nd	##	nd	nd	nd	nd
GV_XG	nd	nd	nd	3	52	nd	401	3	nd	522	426	nd	nd	nd	nd	21	nd	nd	nd	nd
GB_T	nd	nd	nd	nd	1023	nd	129	16	1013	nd	72	nd	nd	nd	nd	nd	nd	nd	nd	nd
GB_XG	nd	nd	nd	nd	33	nd	6	1	27	nd	3	nd	nd	nd	nd	nd	nd	nd	nd	nd
GM_T	nd	nd	nd	nd	nd	nd	nd	nd	nd	nd	nd	nd	44901	nd	nd	nd	nd	nd	nd	nd
GM_XG	nd	nd	nd	nd	nd	nd	nd	nd	nd	nd	nd	nd	1899	nd	nd	nd	nd	nd	nd	nd
GA_T	nd	nd	nd	nd	8	116	78	20	nd	nd	500	nd	nd	nd	nd	nd	nd	nd	nd	nd
GA_XG	nd	nd	nd	nd	nd	5	4	nd	nd	nd	14	nd	nd	nd	nd	nd	nd	nd	nd	nd
GEC_T	nd	nd	nd	nd	950	nd	48	nd	nd	nd	67	nd	nd	nd	nd	nd	nd	nd	nd	nd
GEC_XG	nd	nd	nd	nd	54	nd	3	nd	nd	nd	nd	nd	nd	nd	nd	nd	nd	nd	nd	nd
GCU_T	nd	nd	nd	nd	nd	nd	nd	nd	nd	nd	nd	nd	45722	nd	nd	nd	nd	nd	nd	nd
GCU_XG	nd	nd	nd	nd	nd	nd	nd	nd	nd	nd	nd	nd	2191	nd	nd	nd	nd	nd	nd	nd
